# Post-Marketing Pharmacovigilance of Canakinumab from the FDA Adverse Event Reporting System (FAERS)

**DOI:** 10.3390/ph18010114

**Published:** 2025-01-16

**Authors:** Weidong Zhang, Yunzhou Chen, Zeyu Yao, Mengling Ouyang, Minghui Sun, Shupeng Zou

**Affiliations:** 1 Department of Pharmacy, Tongji Hospital, Tongji Medical College, Huazhong University of Science and Technology, Wuhan 430030, China; zz2495574490@163.com (W.Z.);; 2 Faculty of Engineering, The Hong Kong Polytechnic University, Hong Kong, China

**Keywords:** FAERS, adverse events, pharmacovigilance, canakinumab, data mining

## Abstract

**Background:** Canakinumab, a humanized anti-IL-1β monoclonal antibody, is known for its ability to suppress IL-1β-mediated inflammation. However, continuous monitoring of its safety remains essential. Thus, we comprehensively evaluated the safety signals of canakinumab by data mining from FAERS. **Methods:** We used a disproportionate analysis to quantify canakinumab-related adverse events (AEs) using four algorithms. Clinical prioritization of the detected signals was assessed with a semiquantitative score method. Serious and non-serious outcomes were compared by statistical methods. Additionally, a stratification analysis of serious infections was conducted at the system organ class (SOC) level. **Results:** A total of 28,496 canakinumab-related AEs were collected, and 71 suspicious signals detected. Among these, 19 preferred terms (PTs) were identified as unexpected signals, including deafness, appendicitis, brain oedema, cushingoid, cellulitis, and papilledema. Of the AEs, 16 were more likely reported as serious outcomes, such as pneumonia, abdominal pain, deafness, and infection. Based on clinical priority score, 44 PTs were classified as weak, 27 as moderate, and none as strong. Furthermore, 30 PTs demonstrated a high level of evidence, primarily derived from FDA prescribing information, randomized controlled trials, and systematic reviews. Stratification analysis of infections and infestations (serious outcomes) revealed a stronger association of severe infections with canakinumab in older or heavier individuals. All positive signals followed an early failure pattern, with the incidence of canakinumab-associated AEs decreasing over time. **Conclusions:** We found that most of the suspicious signals were associated with infections. More attention should be paid to serious infections, particularly in males, individuals aged ≥60 years, or those weighing >100 kg, who demonstrated the highest risk of serious infections.

## 1. Introduction

Cryopyrin-associated periodic syndrome (CAPS) is a rare hereditary autoinflammatory disease with an incidence of about 1/1,000,000 [1]. CAPS is associated with NLRP3 pathogenic variants, which lead to the hyperactivation of the NLRP3 inflammasome, resulting in the inappropriate release of inflammatory cytokines such as interleukin-1β (IL-1β) [2]. The clinical manifestations include recurrent fever, headache, rash, joint pain, conjunctivitis, and more [3]. CAPS can be divided into three categories: familial cold autoinflammatory syndrome (FCAS), Muckle–Wells syndrome (MWS), and neonatal-onset multisystem inflammatory disease/chronic infantile neurological cutaneous and articular syndrome (NOMID/CINCA) [4]. Prolonged development with CAPS can lead to bone and nerve abnormalities and other severe or even life-threatening outcomes, such as joint deformities, optic nerve damage, hearing loss, and kidney failure [5].

Canakinumab, approved for the treatment of FCAS and MWS, can bind to IL-1β and block its interaction with IL-1β receptors, thereby inhibiting IL-1β-mediated inflammation responses [6,7]. This mechanism allows canakinumab to be used to treat a variety of inflammation-related diseases. In addition to CAPS, canakinumab has been tested in 58 completed or still ongoing studies, such as Still’s disease, Behcet’s disease, urticarial vasculitis, pyoderma gangrenosum, osteoarthritis, heart failure, chronic obstructive pulmonary disease, and sickle cell disease [8]. Like active Still’s disease, previous studies have associated inflammatory diseases with single nucleotide polymorphisms in IL-6, IL-18, macrophage inhibitory factor (MIF), and serum amyloid A [9,10,11]. A randomized controlled trial showed that canakinumab was superior to triamcinolone acetonide in acute gout and reduced the risk of new gout attacks by 62% [12]. Compared with other IL-1 inhibitors (anakinra, an IL-1 antagonist, and rilonacept, an IL-1 trap fusion protein), the long half-life and good tolerance of canakinumab also help to prolong its anti-inflammatory effect [13,14]. In 2015, Cassyanne L Aguiar et al. reported that a 2-year-old systemic juvenile idiopathic arthritis (JIA) patient tolerated canakinumab safely after allergic reaction to anti-IL-1 therapy (anakinra) [15]. Moreover, because it results in a 35–40% reduction in IL-6 and high-sensitivity C-reactive protein (hsCRP), which are related to cardiovascular events, canakinumab is also considered to have therapeutic potential for atherosclerotic disease [16,17]. Svensson et al. found canakinumab (hazard ratio, 0.38 [95% CI, 0.15–0.96]) reduced the risk of major adverse cardiovascular events (MACEs) in patients with clonal hematopoiesis of indeterminate potential (CHIP) [18]. Zhou et al. reported that the launch price of drugs may not significantly influence the reimbursement decision in China, and the higher clinical value should be the focus of the medical insurance reimbursement policy, especially for drugs supported by randomized controlled trials [19].

Primary inflammasome proteins, such as NLRP3, NLRC4, NLRP1, and AIM2, are also associated with human malignancies via the microenvironment of autologous immunity [20]. Canakinumab, as an IL-1β inhibitor, is also used in combination with other antineoplastic agents to treat non-small cell lung cancer (NSCLC). However, several multicenter, randomized, double-blind trials found that adding canakinumab to docetaxel or cisplatin-based chemotherapy or first-line pembrolizumab did not provide additional benefit for patients with NSCLC [21,22,23]. No unexpected signals were observed for canakinumab in these studies.

Common adverse events caused by canakinumab included infection, fever, rash, arthralgia, urinary tract infection, and more [13]. Serious adverse events (SAEs), such as opportunistic infection, malignant tumor or death, were not observed in pediatric patients with familial Mediterranean fever (FMF) [24]. However, a long-term safety study in Germany reported that a CINCA/NOMID patient with amyloidosis died of septic shock while receiving canakinumab [25]. In a long-term clinical study of canakinumab, Krause et al. reported seven cases of severe AEs, including pneumonia with transient hemiplegia, sepsis with atypical mycobacterium disease, and more [26]. A long-term safety cohort study of canakinumab in patients with CAPS showed that when receiving higher than the recommended starting dose (SD), adverse events (AEs) commonly manifested as serious infections and serious adverse drug reactions [7]. Despite canakinumab playing an important role in clinics because of its strong anti-inflammatory effect and wide range of indications, the continuous pharmacovigilance of AEs cannot be ignored. Feng et al. developed a machine learning model basing a drug repurposing recommendation model called MRNDR (Multi-head attention-based Recommendation Network for Drug Repurposing) to explore the pharmaceutical properties of diverse drug candidates [27].

In our study, we utilized the U.S. Food and Drug Administration (FDA) adverse event reporting system database FAERS (an independent adverse events reporting system) to evaluate the safety of canakinumab after marketing using the disproportionate analysis method. Then, the characteristics of AEs related to canakinumab were detected by the analyses of stratifications, time of onset, and serious vs. non-serious. Notably, the serious outcomes included death, threat to life, hospitalization, disability, and other serious events [28].

## 2. Results

### 2.1. Descriptive Analysis

After data mining from 2009 (Q3, quarter) to 2024 (Q3), a total of 49,488,841 AEs were collected from FAERS, among which 28,496 AEs were associated with canakinumab. The detailed clinical features of canakinumab-related AEs are shown in Table 1. Of all the AEs, the proportion of females (16,207, 56.87%) was higher than that of males (10,693, 37.52%). The median age of patients with canakinumab from FAERS was 18 years (9, 48), as a median (Q1, Q3 [quartile 1, quartile 3]). The United States had the largest number of AE reports (16,027, 56.24%), followed by Canada (3273, 11.49%) and Japan (2740, 9.62%), respectively. Because the FAERS database was established by the U.S. FDA, most of the reports came from the United States. The top four indications were Still’s disease (1997, 7.01%), cryopyrin periodic syndrome (1289, 4.52%), Juvenile idiopathic arthritis (1134, 3.98%), and cardiovascular event prophylaxis (741, 2.60%). Hospitalization among these outcomes was the most frequently reported serious consequence (8698, 30.52%). It was worth noting that 1604 cases reportedly resulted in death, accounting for 5.63%. In terms of reporting years, the number of reports has increased year by year in the past five years, and the number of reports reached 4032 cases (14.15%) in 2023.

### 2.2. Disproportionality Analysis

As shown in Table 2, the disproportionality analysis of canakinumab at the system organ class (SOC) level was observed from FAERS. The AEs induced by canakinumab were statistically significant in 10 organ systems, meeting one of four algorithms in Supplementary Table S2, such as: congenital, familial, and genetic disorders (*n* = 316, ROR [reporting odds ratio] 3.78, 95% CI: 3.38–4.22); infections and infestations (*n* = 3430, ROR 2.45, 95% CI: 2.36–2.53); injury, poisoning, and procedural complications (*n* = 4119, ROR 1.63, 95% CI: 1.58–1.69); immune system disorders (*n* = 471, ROR 1.49, 95% CI: 1.36–1.64); musculoskeletal and connective tissue disorders (*n* = 1990, ROR 1.35, 95% CI: 1.29–1.41); ear and labyrinth disorders (*n* = 173, ROR 1.40, 95% CI: 1.20–1.62); and respiratory, thoracic, and mediastinal disorders (*n* = 1597, ROR 1.17, 95% CI: 1.12–1.23).

As shown in Table 3, 71 different PTs and 16 corresponding SOCs of canakinumab conformed to the four algorithms. It is worth noting that, among these, 19 PTs were classified as unexpected AEs, which were unlisted in the package insert, such as deafness (ROR, 3.02), cushingoid (ROR, 7.87), appendicitis (ROR, 5.60), brain oedema (ROR, 3.02), pleurisy (ROR, 5.93), erysipelas (ROR, 7.65), and more. In 71 PTs, 36 PTs were associated with infections approximately.

### 2.3. Clinical Prioritization of the Suspicious PTs

In Table 3, according to the designated medical event (DME) and important medical event (IME) lists from the European Medicines Agency (EMA), we used a semiquantitative score scale to assess the clinical priorities of significant PTs [28]. In Supplementary Table S1, the significant PTs with weak, moderate, or strong clinical priority depended on the score between 0 and 4, 5 and 7, or 8 and 10, respectively [29]. We aimed to distinguish the special PTs and identified 44 PTs, 27 PTs, and 0 PTs as weak, moderate, and strong. Significantly, the moderate PTs included deafness, papilledema, pneumonia, gastroenteritis, appendicitis, erysipelas, meningitis, Behcet’s syndrome, and more. Of the PTs, 30 showed a strong level of evidence with “++”, including varicella, impetigo, rash, rhinitis pharyngitis, tonsillitis, pharyngitis streptococcal, oropharyngeal pain, influenza, nasopharyngitis, cough, pneumonia, arthralgia, and more.

### 2.4. Stratification Analysis

**Figure 1:**
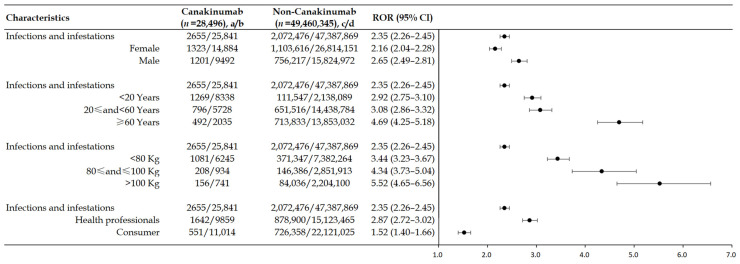
Stratification analysis of canakinumab-related AEs of infections and infestations (serious outcomes) at SOC level. SOC: system organ class. ROR: reporting odds ratio. CI: confidence interval. *n*: number of cases of total AEs associated with the target drug. a/c: number of cases with suspected AEs associated with the target drug. b/d: number of cases without suspected AEs (i.e., total AEs excluding suspected ones) associated with the target drug. SOC of infections and infestations (serious outcomes) was assessed separately by sex, age, body weight, and reporter type.

In the boxed warning of canakinumab, canakinumab has been associated with an increased incidence of serious infections. We used four different layering strategies to analyze the association between serious infection and canakinumab. As shown in Figure 1, after evaluating infected and invaded SOC (serious) by sex, age, body weight, and reporter type, the lower limit of ROR was greater than 1, indicating that canakinumab has a strong correlation with serious infections in different stratified subgroups. And, in age and body weight groups, we could find that as age and weight increased, the ROR values of the different groups also increased generally, with the highest ROR in different subgroups as follows: mela (ROR 2.65, 95% CI: 2.49–2.81), ≥60 years (ROR 4.69, 95% CI: 4.25–5.18), >100 kg (ROR 5.52, 95% CI: 4.65–6.56), and health professionals (ROR 2.87, 95% CI: 2.72–3.02).

### 2.5. Serious vs. Non-Serious Cases

In an analysis of serious vs. non-serious cases, as shown in Table 4, there were significant statistical differences in sex (female and male), age (22 vs. 14 years, *p* < 0.001), and body weight (55 vs. 58 kg, *p* < 0.001) between severe and non-severe AEs associated with canakinumab. Among the 37 kinds of AEs associated with canakinumab, at least 16 AEs (such as pneumonia, abdominal pain, deafness, infection, pneumonia, and more) were more likely to be reported as severe AEs, *p* < 0.05. It was worth noting that all 40 cases of eosinophilia and systemic symptoms (AE) were severe.

### 2.6. Time to Onset Analysis

We gathered the onset time of canakinumab-related adverse events from the FAERS database. The results of the time to onset (TTO) and Weibull shape parameter (WSP) analysis for different priority signals in one year are shown in Table 5. The median TTOs of moderate and weak signals associated with canakinumab were 57.0 (IQR, 14–160) days and 53.5 (IQR, 10–144) days. According to the WSP analysis, the shape parameter *β* and its upper limit of the 95% CI were both <1, suggesting that there were early types of failures in these clinical priority signals.

## 3. Discussion

For the first time, we systematically and comprehensively evaluated the relevant AEs reports of canakinumab using the FAERS database. Previous studies on the safety of canakinumab have mostly focused on clinical trials or serious AEs such as macrophage activation syndrome [30,31].

The proportion of canakinumab-related adverse event reports has increased year by year in the past five years. It rose from 2266 cases (7.95%) in 2019 to 4032 cases (14.15%) in 2023. We found the sales of canakinumab were increasing year by year in the PharmaCompass database (https://www.pharmacompass.com/sales-forecast/canakinumab, accessed on 31 November 2024). In 2024 Q1–Q3 (3252 cases, 11.41%), the reporting rate of canakinumab-related AEs seemed to be stabilizing. The median age of adverse events after using canakinumab was 18 years (9–48), and the incidence of female patients was higher than that of male patients (56.87% vs 37.52%). For Still’s disease or JIA, some reports showed the incidence in females is higher than that of males [32,33]. In addition, compared with severe cases and non-severe cases, sex (*p* < 0.001), patient age (*p* < 0.001), and body weight (*p* < 0.001) might be related to the increased risk of serious AEs caused by canakinumab. Our disproportion analysis showed that the significant SOCs of canakinumab were mainly concentrated in congenital, familial, and genetic disorders; infections and infestations; general disorders and administration site conditions; ear and labyrinth disorders; musculoskeletal and connective tissue disorders; respiratory, thoracic, and mediastinal disorders; and immune system disorders.

In previous reports, common adverse reactions after using canakinumab included pneumonia, tuberculosis, urinary tract infection, histoplasmosis, invasive fungal infection, and gastroenteritis [24,34]. Some adverse reactions of canakinumab reported in clinical trials were confirmed by our study, such as splenomegaly, lymphadenitis, pericarditis, hepatomegaly, meningitis, and rhinorrhea. A multicenter national study of adults and children from France showed that more than half of patients presented at least one adverse event after receiving canakinumab treatment, 17% had mild respiratory infections, and 9% had hepatotoxicity [35]. They found that injection site reactions and liver toxicity were significantly more frequent in children than in adults [35]. An open label III multicenter study also showed that SAEs were more frequent in pediatric patients than in the entire cohort (12.8% vs. 10.8%), such as pneumonia, worsening of headache, and serum sickness syndrome [36]. Other IL-1 inhibitors, such as anakinra, also reported similar adverse events to canakinumab [37]. The different suspicious PTs (71 PTs conformed to four algorithms, 19 PTs in new) in our study were deafness, appendicitis, brain oedema, cushingoid, cellulitis, papilledema, and so on. In addition, some AEs could lead to serious consequences, and our study showed that there were significant statistical differences between severe and non-severe cases (*p* < 0.001) in many PTs, like arthralgia, malaise, rash, infection, and more. In our analysis of severe vs. non-severe cases, 37 PTs showed a significant difference. 16 AEs (such as pneumonia, abdominal pain, deafness, infection, pneumonia, and more) were more likely to be reported as severe AEs, *p* < 0.05. Krause et al. reported seven cases of severe canakinumab during a long-term clinical study of AEs, including a 68-year-old patient with pneumonia with transient hemiplegia and another patient with sinusitis with fever and anemia after 8 months of canakinumab treatment, who died of sepsis with atypical mycobacterium disease 10 weeks later [26]. Yamasaki et al. reported for the first time a case of inflammatory bowel disease with gastrointestinal symptoms and arthritis during the treatment of CAPS with canakinumab [38]. According to the TTO analysis over one year, we also found 70% of cases occurred within the first four months after canakinumab treatment. The TTO analysis also displayed signal characteristics (moderate and weak) akin to early failure type, which suggested a gradual reduction in the risk of canakinumab-related adverse event occurrence over time.

In a long-term safety and effectiveness study of canakinumab, serious infections were more common in patients receiving higher than the recommended starting dose (SD) within a CAPS cohort [7]. Our stratification analysis of serious infections (serious outcomes) revealed that the males, aged ≥60 years, or weighing >100 kg showed the strongest association with serious infections. A systematic review found that a higher incidence of macrophage activation syndrome (MAS) secondary to infection had been noted in patients treated with canakinumab than in those treated with anakinra [39]. A randomized controlled trial of canakinumab for the treatment of autoinflammatory recurrent fever syndromes showed that the most frequently reported adverse events were infections (313.5 per 100 patient-years), with a few being serious infections (13.7 per 100 patient-years) [40]. Another long-term study of the efficacy and safety of canakinumab showed that no association was observed between increased cumulative dose of canakinumab and the occurrence of serious infections or SAEs [41]. Coadministration of canakinumab with tumor necrosis factor receptor (TNF) inhibitors was not recommended in the FDA prescribing information, because this might increase the risk of serious infections. As expected, canakinumab treatment led to a small but statistically significant increase in deaths due to infection and sepsis as a result of inhibiting the innate host defense mechanisms [42].

Canakinumab can reduce the risk of cardiovascular disease by 15%, which is the first direct proof that anti-inflammatory drugs can reduce the incidence of cardiovascular disease [42]. In a randomized, double-blind trial published in the Lancet by Ridker et al., 300 mg of canakinumab reduced the risk of lung cancer by 67% and 77%, respectively [43]. The results of these two studies are hopeful. Since the increase in IL-1β is closely related to tumorigenesis and invasiveness, IL-1 inhibitors are also a potential anticancer method [44]. However, the anti-inflammatory properties of canakinumab blocking the IL-1 pathway may weaken the immune response of patients and increase the risk of opportunistic infection [45]. If canakinumab is approved for the secondary prevention of cardiovascular events, it cannot be overlooked that it may increase the risk of opportunistic infection. The TTO and WSP analysis revealed that the majority of adverse events occurred within the initial four months after canakinumab therapy, displaying characteristics of all signals akin to early failure type, and suggesting a gradual reduction in the risk of canakinumab-related adverse event occurrence over time.

Our disproportionate analysis also found 19 new unexpected adverse events related to canakinumab that were not reported in some drug instructions, including appendicitis, ear infection, papilledema erysipelas, subcutaneous abscess, cellulitis, pleurisy, COVID-19, cushingoid, intracranial pressure increase, blood fibrinogen decrease, blood lactate dehydrogenase increase, brain oedema, drug reaction with eosinophilia and systemic symptoms, rhinovirus infection, infectious mononucleosis, Epstein–Barr virus infection, ear pain, and deafness. These new PTs should be marked as security alerts by clinicians. TTO analysis showed that most AEs occurred within 1 month after canakinumab treatment. However, the potential mechanism of action between drugs and AEs has not been fully explored, and further clinical research is needed.

It is undeniable that our research also has some limitations, which are related to the inherent nature of the FAERS database. Firstly, as the FAERS database is a reporting platform for the public, the reporter may not have medical expertise, so the completeness and accuracy of the report cannot be guaranteed. Some AEs might be caused by diseases. Secondly, we cannot clarify the causal relationship between target drugs and adverse events, because the disproportion analysis is only statistically significant. Thirdly, due to the missing records, we were unable to consider other factors in the stratified analysis, such as comorbidities and doses administered. Therefore, we were also unable to adjust for multiple comparisons in our analysis, which might falsely inflate signals. Additionally, the effects of potential drug–drug interactions and changes in treatment regimens and detection methods on adverse events are not included in the data analysis, and further clinical trials and cohort studies are necessary to verify these results.

## 4. Materials and Methods

### 4.1. Study Design

Our retrospective pharmacovigilance study used a disproportionate analysis to assess whether there might be a correlation between canakinumab and suspicious AEs. Our study extracted American Standard Code for Information Exchange (ASCII) data from the fourth quarter (Q3) of 2009 to Q3 of 2024 from the FAERS database. The data files of FAERS were available from the official website (https://fis.fda.gov/extensions/FPD-QDE-FAERS/FPD-QDE-FAERS.html, accessed on 12 November 2024).

### 4.2. Data Mining

**Figure 2:**
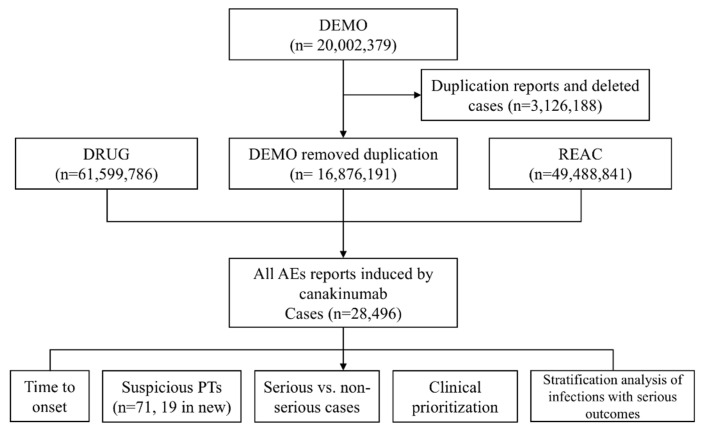
The process of canakinumab-associated adverse events from the Food and Drug Administration Adverse Event Reporting Database (FAERS). DEMO: demographic file; REAC: reaction file; AEs: adverse events; PTs: preferred terms.

All data from this retrospective analysis were derived from the official FAERS website and then further processed through SAS software (version 9.4). Because repeated reports are inevitable, we deduplicated the data based on the unique ID (primaryid) in the demographic file [DEMO] [28]. We used the common name “canakinumab” and the trade name “Ilaris” as the target drugs, and selected primary suspected (PS) as the drug role code in the drug file [DRUG]. Adverse events of canakinumab in FAERS reports are encoded using the PTs in Medical Dictionary for Regulatory Activities (MedDRA). Subsequently, the clinical features of the report (sex, age, body weight, reporting area, indications, and results) are descriptively analyzed in Table 1. Severe outcomes refer to death, hospitalization, threat to life, disability, and other serious outcomes [29]. Notably, because one adverse event might correspond to multiple serious outcomes, the number of serious consequences might be higher than the non-serious outcomes. The process of our study (including data identification, extraction, processing, and outcomes) is shown in Figure 2. We also used a semiquantitative score method to access the clinical priority by five different features in Supplementary Table S1, including the number of AE reports, ROR value, mortality proportion, IME or DME, and relevant evidence evaluation [29].

### 4.3. Time to Onset Analysis

The TTO of adverse events used the following formula:

Time-to-onset = Event onset date (EVENT_DT) − Therapy start date (START_DT),

We deleted reports with input errors ((EVENT_DT) is earlier than (START_DT)) and missing dates [31]. The WSP test was used to evaluate TTO data characteristics. The scale parameter *α* of the Weibull distribution determines the scale of the distribution function, and the shape parameter β determines the shape of the distribution function. When the shape parameter β < 1 and its 95% CI < 1, the AE is considered to decrease over time (early failure-type profile); when the shape parameter β is equal to or close to 1 and its 95% CI includes a value of 1, this indicates that the AE does not change over time (random failure-type profile); and when the shape parameter β > 1 and its 95% CI excludes a value of 1, this indicates that the AE increases over time (wear-out failure-type profile) [28].

### 4.4. Statistical Analysis

In our study, a disproportionate analysis used four different algorithms to reduce the systematic bias, including reporting odds ratio, proportional report ratio (PRR), the Bayesian confidence propagation neural network (BCPNN), and the multi-item gamma Poisson shrinker (MGPS) [46,47]. The calculation methods and criteria of the algorithms are shown in Supplementary Table S2. Among them, PTs conformed to the criteria of four algorithms, which were suspected to be positive signals [48]. The higher value of the algorithms showed a stronger association between AEs and the target drug [49]. In the comparison of severe and non-severe outcomes, Pearson chi-square or Fisher exact tests were used for proportion comparison, and the Mann–Whitney U test was used for continuous non-normal distribution data (age and body weight) [50,51]. When *p* < 0.05, it is considered to be statistically significant. We also performed a stratification analysis of infections and infestations (serious outcomes) at the SOC level.

## 5. Conclusions

Our drug alert analysis is based on a large sample of real-world safety data from the FAERS database, providing a comprehensive and systematic evaluation of the safety of canakinumab. Among the 28,496 reports related to canakinumab, 71 suspected PTs were identified, including 19 newly detected PTs not listed in the drug labeling. Most AEs occurred within four months of initiating canakinumab treatment. We found that most of the suspicious signals were associated with infections. Serious infections warrant attention, particularly in males, individuals aged ≥60 years, and those weighing >100 kg, who showed the strongest association with these outcomes. Although our research provided valuable insights into the safety of canakinumab, long-term clinical studies are needed to further verify these results.

## Figures and Tables

**Table 1:** Characteristics of reports from the FAERS database (Q3 2009 to Q3 2024).

		Canakinumab *n* (%)
Gender	Female	16,207 (56.87)
	Male	10,693 (37.52)
	NA	1596 (5.61)
Age (Years)	Median (IQR)	18 (9, 48)
Time to onset ^a^ (Days)	Median (IQR)	54.5 (10–147)
Reported Countries	United States	16,027 (56.24)
	Canada	3273 (11.49)
	Japan	2740 (9.62)
	Germany	1303 (4.57)
	United Kingdom	632 (2.20)
Reporters	Consumer	11,565 (40.58)
	Physician	9117 (31.99)
	Pharmacist	4973 (17.45)
	Other health professional	2384 (8.37)
Indications	Still’s disease	1997 (7.01)
	Cryopyrin periodic syndrome	1289 (4.52)
	Juvenile idiopathic arthritis	1134 (3.98)
	Cardiovascular event prophylaxis	741 (2.60)
	Pyrexia	680 (2.39)
Outcomes	Hospitalization—Initial or Prolonged	8698 (30.52)
	Other Serious Events	6945 (24.37)
	Death	1604 (5.63)
	Disability	432 (1.52)
	Threat to Life	242 (0.85)
Reporting year (Near 6 years)	2024 (Q1–Q3)	3252 (11.41)
	2023	4032 (14.15)
	2022	3087 (10.83)
	2021	2745 (9.63)
	2020	2362 (8.29)
	2019	2266 (7.95)

^a^: the time to adverse event onset in one year. N: the number of reports. Q: quarter. IQR: interquartile range (Q1, Q3).

**Table 2:** The signal strength of adverse events of canakinumab at the system organ class (SOC) level in the FAERS database.

System Organ Class (SOC)	Case Reporting	ROR (95% CI)	PRR (χ^2^)	IC (IC_025_)	EBGM (EBGM_05_)
Congenital, familial, and genetic disorders	316	3.78 (3.38–4.22) ^a^	3.74 (3496.67) ^a^	1.90 (1.70) ^a^	3.74 (3.41) ^a^
Infections and infestations	3430	2.45 (2.36–2.53) ^a^	2.27 (252,944.11) ^a^	1.18 (1.14) ^a^	2.27 (3.20) ^a^
Injury, poisoning, and procedural complications	4119	1.63 (1.58–1.69) ^a^	1.54 (149,793.00)	0.62 (0.60) ^a^	1.54 (4.50) ^a^
Immune system disorders	471	1.49 (1.36–1.64) ^a^	1.48 (1554.45)	0.57 (0.52) ^a^	1.48 (4.38) ^a^
General disorders and administration site conditions	6522	1.39 (1.36–1.43) ^a^	1.30 (182,180.45)	0.38 (0.37) ^a^	1.30 (6.27) ^a^
Musculoskeletal and connective tissue disorders	1990	1.35 (1.29–1.41) ^a^	1.32 (16,083.22)	0.40 (0.38) ^a^	1.32 (1.27)
Ear and labyrinth disorders	173	1.40 (1.20–1.62) ^a^	1.39 (155.71)	0.48 (0.41) ^a^	1.39 (1.23)
Respiratory, thoracic, and mediastinal disorders	1597	1.17 (1.12–1.23) ^a^	1.16 (3463.49)	0.22 (0.21) ^a^	1.16 (1.12)
Blood and lymphatic system disorders	510	1.05 (0.96–1.14)	1.05 (34.17)	0.07 (0.06) ^a^	1.05 (5.97) ^a^
Hepatobiliary disorders	277	1.05 (0.94–1.19)	1.05 (12.69)	0.07 (0.07) ^a^	1.05 (2.95) ^a^

^a^ Indicates statistically significant signals in the algorithm. ROR: reporting odds ratio. CI: confidence interval. PRR: proportional reporting ratio. χ^2^: chi-squared. IC: information component of BCPNN. IC_025_: the lower limit of 95% CI of the IC. BCPNN: Bayesian confidence propagation neural network. EBGM: empirical Bayesian geometric mean. EBGM_05_: the lower limit of 95% CI of EBGM.

**Table 3:** Signal strength and clinical priority assessing results of preferred terms (PTs) of canakinumab from FAERS database.

SOC	Preferred Terms (PTs)	Cases	ROR (95% CI)	IC (IC_025_)	Death (*n*)	IME/ DME	Relevant Evidence Evaluation	Priority Level (Score)
Blood and lymphatic system disorders	Lymphadenopathy	77	4.88 (3.90–6.11)	2.28 (1.82) ^b^	7	NA	++	Moderate (5)
	Splenomegaly	24	4.55 (3.05–6.80)	2.18 (1.46)	4	NA	+	Weak (3)
	Lymphadenitis	10	9.51 (5.11–17.70)	3.24 (1.74) ^b^	0	NA	+	Weak (4)
Cardiac disorders	Pericarditis	28	4.55 (3.14–6.59)	2.18 (1.50)	0	IEM	+	Weak (4)
Ear and labyrinth disorders	Deafness	36	3.02 (2.18–4.19)	1.59 (1.15)	0	DEM	-	Weak (4)
	Ear pain	26	2.89 (1.97–4.25)	1.53 (1.04)	0	NA	-	Weak (2)
Gastrointestinal disorders	Abdominal pain	242	2.31 (2.04–2.62)	1.20 (1.06)	11	NA	++	Moderate (5)
	Gastroenteritis	57	8.95 (6.90–11.61)	3.15 (2.43) ^b^	0	NA	++	Moderate (6)
	Mouth ulceration	30	3.23 (2.26–4.62)	1.69 (1.18)	2	NA	+	Weak (3)
	Gastroenteritis viral	29	3.45 (2.40–4.97)	1.78 (1.24)	0	NA	+	Weak (3)
General disorders and administration site conditions	Malaise	671	3.18 (2.95–3.44)	1.65 (1.52) ^b^	11	NA	+	Weak (4)
	Condition aggravated	638	4.98 (4.60–5.39)	2.29 (2.11) ^b^	12	NA	+	Weak (4)
	Illness	242	6.07 (5.35–6.89)	2.59 (2.28) ^b^	3	NA	+	Moderate (5)
	Inflammation	110	4.86 (4.03–5.87)	2.27 (1.89) ^b^	3	NA	++	Moderate (5)
	Disease recurrence	57	2.52 (1.94–3.26)	1.33 (1.02)	2	NA	+	Weak (4)
	Concomitant disease aggravated	48	15.44 (11.62–20.52)	3.93 (2.96) ^b^	4	NA	+	Weak (4)
	Symptom recurrence	21	8.32 (5.42–12.78)	3.05 (1.99) ^b^	0	NA	+	Weak (4)
	Concomitant disease progression	17	19.64 (12.18–31.69)	4.28 (2.65) ^b^	3	NA	+	Weak (4)
Hepatobiliary disorders	Hepatomegaly	28	6.87 (4.74–9.96)	2.77 (1.91) ^b^	5	NA	+	Weak (4)
Immune system disorders	Decreased immune responsiveness	22	4.77 (3.14–7.26)	2.25 (1.48)	3	NA	+	Weak (3)
	Immunosuppression	13	3.43 (1.99–5.92)	1.78 (1.03)	0	IEM	++	Moderate (5)
Infections and infestations	Infection	163	2.47 (2.12–2.89)	1.30 (1.11)	12	NA	++	Moderate (5)
	Viral infection	67	4.55 (3.58–5.79)	2.18 (1.72) ^b^	4	NA	++	Moderate (5)
	Ear infection	66	5.37 (4.21–6.84)	2.42 (1.90) ^b^	0	NA	-	Weak (4)
	Conjunctivitis	28	3.45 (2.38–4.99)	1.78 (1.23)	0	NA	++	Weak (4)
	Abscess	26	3.56 (2.43–5.24)	1.83 (1.25)	0	NA	++	Weak (4)
	Appendicitis	23	5.60 (3.72–8.43)	2.48 (1.65) ^b^	0	IEM	-	Weak (4)
	Streptococcal infection	22	9.48 (6.23–14.42)	3.24 (2.13) ^b^	2	NA	+	Weak (4)
	Otitis media	19	12.97 (8.26–20.37)	3.69 (2.35) ^b^	1	NA	++	Moderate (5)
	Epstein–Barr virus infection	18	6.70 (4.21–10.64)	2.74 (1.72) ^b^	1	NA	-	Weak (3)
	Meningitis	17	5.86 (3.64–9.43)	2.55 (1.58) ^b^	0	IEM	+	Moderate (5)
	Coronavirus infection	15	4.41 (2.65–7.31)	2.14 (1.29)	2	NA	++	Weak (4)
	Infectious mononucleosis	11	11.00 (6.08–19.89)	3.45 (1.91) ^b^	0	NA	-	Weak (3)
	Rhinovirus infection	10	6.27 (3.37–11.66)	2.64 (1.42)	1	NA	-	Weak (3)
Injury, poisoning, and procedural complications	DRA with ESS	40	3.28 (2.40–4.47)	1.71 (1.25)	4	DEM	-	Weak (4)
	Brain oedema	16	3.02 (1.85–4.94)	1.59 (0.98)	9	IEM	-	Moderate (5)
Investigations	C-reactive protein increased	109	7.10 (5.88–8.57)	2.82 (2.33) ^b^	9	NA	++	Moderate (6)
	Serum ferritin increased	51	22.06 (16.73–29.08)	4.44 (3.37) ^a^	5	NA	+	Moderate (5)
	Body temperature increased	45	5.07 (3.78–6.79)	2.34 (1.74) ^b^	0	NA	++	Moderate (5)
	SARS-CoV-2 test positive	43	6.10 (4.52–8.24)	2.60 (1.93) ^b^	0	NA	+	Weak (4)
	Transaminases increased	34	3.40 (2.43–4.76)	1.76 (1.26)	1	NA	++	Weak (4)
	Inflammatory marker increased	31	13.51 (9.48–19.23)	3.74 (2.63) ^b^	0	NA	+	Weak (4)
	Blood lactate dehydrogenase increased	30	4.88 (3.41–6.98)	2.28 (1.59) ^b^	4	NA	-	Weak (2)
	Lymphocyte count decreased	29	3.37 (2.34–4.86)	1.75 (1.22)	1	NA	++	Weak (4)
	ESR	27	5.99 (4.10–8.73)	2.58 (1.77) ^b^	2	NA	+	Weak (4)
	Body temperature decreased	18	3.95 (2.49–6.27)	1.98 (1.25)	0	NA	++	Weak (4)
	Blood fibrinogen decreased	9	20.37 (10.56–39.31)	4.33 (2.25) ^b^	2	NA	-	Weak (2)
Musculoskeletal and connective tissue disorders	Arthralgia	495	2.61 (2.38–2.85)	1.37 (1.25)	6	NA	++	Moderate (5)
Nervous system disorders	Intracranial pressure increased	12	4.87 (2.76–8.58)	2.28 (1.29)	0	IEM	-	Weak (3)
Psychiatric disorders	Cushingoid	11	7.87 (4.35–14.23)	2.97 (1.64) ^b^	0	NA	-	Weak (3)
Respiratory, thoracic, and mediastinal disorders	Pneumonia	333	2.25 (2.02–2.51)	1.16 (1.04)	45	IEM	++	Moderate (6)
	Cough	296	2.29 (2.04–2.56)	1.18 (1.06)	9	NA	++	Moderate (5)
	COVID-19	257	2.88 (2.55–3.26)	1.52 (1.34)	24	NA	-	Weak (3)
	Nasopharyngitis	231	2.66 (2.33–3.02)	1.40 (1.23)	5	NA	++	Moderate (5)
	Influenza	187	3.67 (3.17–4.23)	1.87 (1.62) ^b^	0	NA	++	Moderate (5)
	Oropharyngeal pain	138	3.02 (2.55–3.57)	1.59 (1.34)	3	NA	++	Moderate (5)
	Rhinorrhea	123	4.08 (3.42–4.87)	2.02 (1.69) ^b^	1	NA	+	Weak (4)
	Nasal congestion	85	3.17 (2.56–3.92)	1.66 (1.34)	0	NA	+	Weak (4)
	Upper respiratory tract infection	77	3.59 (2.87–4.50)	1.84 (1.47)	0	NA	++	Moderate (5)
	Pharyngitis streptococcal	50	9.76 (7.39–12.89)	3.28 (2.48) ^b^	2	NA	++	Moderate (5)
	Tonsillitis	38	15.60 (11.33–21.47)	3.95 (2.87) ^b^	0	NA	++	Moderate (5)
	Pharyngitis	35	6.13 (4.39–8.54)	2.61 (1.87) ^b^	0	NA	++	Moderate (5)
	Rhinitis	17	4.70 (2.92–7.57)	2.23 (1.38)	0	NA	++	Weak (4)
	Pleurisy	16	5.93 (3.63–9.69)	2.56 (1.57) ^b^	1	NA	-	Weak (3)
Skin and subcutaneous tissue disorders	Rash	444	2.29 (2.08–2.51)	1.18 (1.07)	5	NA	++	Moderate (5)
	Cellulitis	93	3.93 (3.20–4.82)	1.97 (1.61) ^b^	3	NA	-	Weak (3)
	Subcutaneous abscess	18	7.93 (4.99–12.60)	2.98 (1.88) ^b^	0	NA	-	Weak (3)
	Erysipelas	18	7.65 (4.82–12.16)	2.93 (1.84) ^b^	1	IEM	-	Weak (4)
	Impetigo	14	21.86 (12.90–37.04)	4.43 (2.62) ^b^	0	NA	++	Moderate (5)
	Varicella	12	11.23 (6.36–19.81)	3.48 (1.97) ^b^	0	NA	++	Moderate (5)
Vascular disorders	Papilledema	11	5.74 (3.18–10.38)	2.52 (1.39)	0	IEM	-	Weak (4)

^a^: IC_025_ > 3.0 indicates a strong signal. ^b^: 1.5 < IC_025_ ≤ 3.0 indicates a medium-intensity signal. ROR: reporting odds ratio. CI: confidence interval. IC: information component of BCPNN. IC_025_: the lower limit of 95% CI of the IC. BCPNN: Bayesian confidence propagation neural network. ESR: red blood cell sedimentation. DRA with ESS: drug reaction with eosinophilia and systemic symptoms. IME: important medical event. DME: designated medical event. NA: not applicable (for relevant criteria). *n*: number of cases. ++: AEs are mainly from the FDA Prescribing Information, the Summary of Product Characteristics of canakinumab posted by the MHRA, Phase 2/3 RCTs, or systematic reviews, with biological plausibility. +: AEs are mainly from other clinical trials, observational studies, or case reports/series with potential biological plausibility. -: AEs only emerging from disproportionality analyses (emerging findings of canakinumab-associated PTs from FAERS database).

**Table 4:** Differences in clinical characteristics of serious and non-serious reports (*n* ≥ 30).

	Serious Cases (*n* = 17,955)	Non-Serious Cases (*n* = 10,541)	Statistic	*p*-Value
Gender, *n* (%)				
Female	10,055	6152	15.101 ^b^	<0.001 ^a^
Male	6634	3249	110.006 ^b^	<0.001 ^a^
Age, years (median, IQR)	22 (9–50)	14 (8–30)	−139.68 ^d^	<0.001 ^c^
Weight, kg (median, IQR)	55 (31–76)	58 (29–90)	−3.222 ^d^	0.001 ^c^
Types of AEs, *n* ≥ 30 (%)				
Malaise	246 (1.37)	425 (4.03)	204.663 ^b^	<0.001 ^a^
Condition aggravated	361 (2.01)	277 (2.63)	11.561 ^b^	0.001 ^a^
Arthralgia	264 (1.47)	231 (2.19)	20.233 ^b^	<0.001 ^a^
Rash	228 (1.27)	216 (2.05)	26.297 ^b^	<0.001 ^a^
Pneumonia	330 (1.84)	3 (0.03)	186.728 ^f^	<0.001 ^g^
Cough	177 (0.99)	119 (1.13)	1.324 ^b^	0.250 ^a^
COVID-19	99 (0.55)	158 (1.50)	66.720 ^b^	<0.001 ^a^
Abdominal pain	193 (1.07)	49 (0.46)	29.356 ^b^	<0.001 ^a^
Illness	72 (0.40)	170 (1.61)	115.819 ^b^	<0.001 ^a^
Nasopharyngitis	113 (0.63)	118 (1.12)	19.840 ^b^	<0.001 ^a^
Influenza	81 (0.45)	106 (1.01)	31.321 ^b^	<0.001 ^a^
Infection	127 (0.71)	36 (0.34)	15.626 ^b^	<0.001 ^a^
Oropharyngeal pain	79 (0.44)	59 (0.56)	1.976 ^b^	0.160 ^a^
Rhinorrhea	61 (0.34)	62 (0.59)	9.539 ^b^	0.002 ^a^
Inflammation	87 (0.48)	23 (0.22)	12.253 ^b^	<0.001 ^a^
C-reactive protein increased	99 (0.55)	10 (0.09)	36.325 ^b^	<0.001 ^a^
Cellulitis	91 (0.51)	2 (0.02)	47.105 ^f^	<0.001 ^g^
Nasal congestion	41 (0.23)	44 (0.42)	7.983 ^b^	0.005 ^a^
Lymphadenopathy	59 (0.33)	18 (0.17)	6.140 ^b^	0.013 ^a^
Upper respiratory tract infection	39 (0.22)	38 (0.36)	5.060 ^b^	0.024 ^a^
Viral infection	52 (0.29)	15 (0.14)	6.145 ^b^	0.013 ^a^
Ear infection	30 (0.17)	36 (0.34)	8.746 ^b^	0.003 ^a^
Gastroenteritis	53 (0.30)	4 (0.04)	20.746 ^f^	<0.001 ^g^
Disease recurrence	43 (0.24)	14 (0.13)	3.786 ^b^	0.052 ^a^
Serum ferritin increased	45 (0.25)	6 (0.06)	13.950 ^b^	<0.001 ^a^
Pharyngitis streptococcal	14 (0.08)	36 (0.34)	26.338 ^b^	<0.001 ^a^
Concomitant disease aggravated	47 (0.26)	1 (0.01)	23.660 ^f^	<0.001 ^g^
Body temperature increased	20 (0.11)	25 (0.24)	6.664 ^b^	0.010 ^a^
SARS-CoV-2 test positive	10 (0.06)	33 (0.31)	29.199 ^b^	<0.001 ^a^
Eosinophilia and systemic symptoms	40 (0.22)	0 (0.00)	-	<0.001 ^e^
Tonsillitis	34 (0.19)	4 (0.04)	10.325 ^f^	0.001 ^g^
Deafness	35 (0.19)	1 (0.01)	16.663 ^f^	<0.001 ^g^
Pharyngitis	23 (0.13)	12 (0.11)	0.110 ^b^	0.740 ^a^
Transaminases increased	31 (0.17)	3 (0.03)	10.409 ^f^	0.001 ^g^
Inflammatory marker increased	24 (0.13)	7 (0.07)	2.765 ^b^	0.096 ^a^
Mouth ulceration	23 (0.13)	7 (0.07)	2.403 ^b^	0.121 ^a^
Blood lactate dehydrogenase increased	28 (0.16)	2 (0.02)	10.582 ^f^	0.001 ^g^

The AEs listed above were AEs with significant signal strengths (*n* ≥ 30). ^a^ Proportions were compared using Pearson χ^2^ test. ^b^ The χ^2^ statistic of the Pearson chi-square test. ^c^ Mann–Whitney U test. ^d^ The Z statistic of the Mann–Whitney U test. ^e^ Fisher’s exact test. ^f^ χ^2^ statistic of the Yates’s correction for continuity. ^g^ Proportions were compared using Yates’s correction for continuity. *p*-value < 0.05 was considered statistically significant.

**Table 5:** The results of time to onset analysis for signals with different prioritizations in one year.

Prioritization	TTO (Days)		Weibull Distribution		Failure Type
	Cases (*n*)	Median (IQR)	Scale Parameter α (95% CI)	Shape Parameter β (95% CI)	
Moderate	1160	57.0 (14–160)	102.81 (95.65–109.98)	0.92 (0.87–0.96)	Early failure
Weak	7164	53.5 (10–144)	100.42 (97.43–103.41)	0.87 (0.86–0.90)	Early failure

*n*, number of cases with available time to onset; IQR, interquartile range; TTO, time to onset.

## Data Availability

The datasets generated and analyzed during the current study are available from the corresponding author upon reasonable request. The FAERS database is available at https://fis.fda.gov/extensions/FPD-QDE-FAERS/FPD-QDE-FAERS.html, accessed on 12 November 2024.

## Supplementary Materials

The following supporting information can be downloaded at https://www.mdpi.com/article/10.3390/ph18010114/s1, Supplementary Table S1: A rating scale assessing clinical priority of disproportionality signals. Supplementary Table S2: Summary of major algorithms used for signal detection.
