# Supplementary material for: Post-Marketing Pharmacovigilance of Canakinumab from the FDA Adverse Event Reporting System (FAERS)
Source: Pharmaceuticals (Basel). 2025 Jan 16;18(1):114. doi: 10.3390/ph18010114 (PMC11768236; doi:10.3390/ph18010114)
Supplement: Supplementary file 1 [file pharmaceuticals-18-00114-s001.zip › pharmaceuticals-3386764-supplementary.pdf]

Supplementary Materials

Supplementary Table S1. A rating scale assessing clinical priority of disproportionality signals.

**Assessment items**	**2 points**	**1 point**	**0 point**
Number of target events	>50	10-50	<10
ROR	>5	2-5	1-2
Mortality proportion	>50%	25-50%	<25%
IMEs or DMEs	DME	IME	None
Relevant evidence evaluation	++	+	-

Mortality proportion: percentage of cases in which death was reported as an outcome in the overall case report for a particular adverse event. IMEs and DMEs are developed and updated by EMA (European Medicines Agency, 2022). ++: adverse events (AEs) are mainly from the FDA Prescribing Information, the summary of product characteristics of canakinumab posted by the MHRA, Phase 2/3 RCTs, or systematic reviews, with biological plausibility. +: AEs are mainly from other clinical trials, observational studies, or case reports/series with potential biological plausibility. -: AEs only emerging from disproportionality analyses. AEs, Adverse Events; DMEs, Designated Medical Events; IMEs, Important Medical Events; MHRA, Medicine and Healthcare Products Regulatory Agency; RCTs, Randomized Controlled Trials; ROR, Reporting Odds Ratio.

Supplementary Table S2. Summary of major algorithms used for signal detection.

Algorithms	Equations	Criteria
ROR	ROR = ad/bc<br>95%CI = $e^{\ln(ROR)\pm1.96(1/a+1/b+1/c+1/d)^\wedge 0.5}$	lower limit of 95% CI > 1,<br>N ⩾ 3
PRR	PRR = (a(c + d))/(c(a + b))<br>$\chi^2 = [(ad - bc)^2](a + b + c + d)/[(a + b)(c + d)(a + c)(b + d)]$	PRR ⩾ 2, χ^2 ⩾ 4,<br>N ⩾ 3
BCPNN	IC = $\log_2 a(a + b + c + d)/((a + c)(a + b))$<br>$IC_{025} = e^{\ln(IC)-1.96(1/a+1/b+1/c+1/d)^\wedge 0.5}$	$IC_{025} > 0$
MGPS	EBGM = a(a + b + c + d)/((a + c)(a + b))<br>$EBGM_{05} = e^{\ln(EBGM)-1.64(1/a+1/b+1/c+1/d)^\wedge 0.5}$	$EBGM_{05} > 2$

a: the number of reports with suspected adverse events (AEs) of the suspect drug; b: the number of reports with all other AEs of the suspect drug; c: the number of reports with the suspected AE of all other drugs; d: the number of reports with all other AEs of all other drugs; ROR: reporting odds ratio; CI: confidence interval; N: the number of co-occurrences; PRR: proportional reporting ratio; χ^2: chi-squared; BCPNN: Bayesian confidence propagation neural network; IC: information component; IC_{025}: the lower limit of the 95% two-sided CI of the IC; MGPS: multi-item gamma Poisson shrinker; EBGM: empirical Bayesian geometric mean; $EBGM_{05}$: the lower 95% one-sided CI of EBGM.
